# Fat taxation in India: A critical appraisal of need, public health impact, and challenges in nationwide implementation

**DOI:** 10.15171/hpp.2020.04

**Published:** 2020-01-28

**Authors:** Yuvaraj Krishnamoorthy, Karthika Ganesh, Manikandanesan Sakthivel

**Affiliations:** ^1^Department of Preventive and Social Medicine, Jawaharlal Institute of Postgraduate Medical Education and Research (JIPMER), Puducherry - 605008, India; ^2^National Institute of Epidemiology (NIE), Chennai, Tamil Nadu, India

**Keywords:** Food Legislation, India, Noncommunicable diseases, Taxes

## Abstract

National Nutritional Monitoring Bureau survey (2017) has found that more than half of the adults in India were overweight and obese. To halt this rising epidemic, development of various policy measures has been suggested in National action plan for prevention and control of noncommunicable diseases. One such measure is the introduction of fat tax which is a surcharge or tax placed on food and beverages containing high amounts of fat. Government of India has made various direct budgetary initiatives for boosting the sectors related to the production of items rich in fat, sugars and salt without realizing the potential public health consequences. Hence, increasing the taxes for unhealthy junk foods should encourage the people to take healthier food options which in turn lead to positive impact on health. However, fat taxationfaced several challenges during implementation in countries like Denmark, Hungary, France and United States. Major challenges were the taxation debate, setting tax limit and encroaching into the autonomy rights of people. Evidences have shown that taxation alone cannot bring down the burden of non-communicable diseases but should be combined with measures like subsidies and access to healthy food items, public health education campaigns and programmes.

## Introduction

Over the past decade, there has been an epidemiological transition from communicable to noncommunicable diseases throughout the world (both developed and developing countries). Major non-communicable diseases account for almost 60% of all deaths and 47% of the global burden of diseases. Major share of these deaths occurs in low- and middle-income countries especially China and India.^1,2^ Factors responsible for this transition are behavioral changes like tobacco and alcohol use and sedentary lifestyle practices such as unhealthy diet and physical inactivity. These factors cause overweight and obesity either directly or indirectly which may in turn lead to major noncommunicable diseases.^3^

The World Health Organization (WHO) has reported that more than 1.9 billion adults worldwide were overweight in 2016 of which 650 million had obesity.^4^ Obesity and overweight were once problems in high income countries, but now are common problems in middle and low income countries.

In India, there has been a growing burden of overweight and obesity over the past three decades. National Nutritional Monitoring Bureau survey 2017 on diet and nutritional status of urban population in India found that more than half of the adults were overweight and obese.^5^ On seeing such a rapid rise in the burden of obesity, Government of India (GoI) has set the target of halting the obesity epidemic by 2025 under the ten targets in National Action Plan for Prevention and Control of Noncommunicable diseases. To achieve this target, action points suggested were development and implementation of policy measures to reduce the use of saturated fats and transfats by food producers and processors.^6^

One policy measure that has been tried and tested in many countries was the introduction of fat tax. Fat tax is surcharge or tax placed on food, beverages containing high amounts of fat or on individuals who are overweight.^7^ Several countries have tried and tested the introduction of taxes on particular food items to internalize the negative externalities that are associated with the intake of environmentally unfriendly and unhealthy food products.^8-12^ Imposing the taxes can raise the price of the commodity which in turn leads to fall in the demand of the unhealthy food items. This paper critically analyzes the public health impact and challenges in introduction of fat taxation throughout India.

## Materials and Methods

### Search strategy

We have conducted literature review on study aimed at identifying the public health impact and challenges in introduction of fat taxation at global level with special focus on India. Search engines such as PubMed, Google Scholar and ScienceDirect were used to obtain literature related to fat taxation throughout the world. Search strategy included different terms such as “Fat Tax”, “Fat Taxation”, “Public Health”, “Challenges”, “Food Tax”, and “Food Production Sectors” as keywords. The date range for search was from inception to 2019.

Inclusion criteria for the reports, legislation documents, case studies, original articles, review articles and other related documents considered for the review were:

(1) Written in English language,

(2) Open access documents and reports available on the journal/website,

(3) Dealt with section on fat taxation, its public health impact and challenges in implementation throughout the world with special focus on India. 22 relevant original and review articles were included in the current review.

## Situational analysis of fat production sectors and policy measures

Directorate of vegetable oil, Vanaspati, and fat (DVOF) has reported that total consumption of the edible oil from both imported and domestic sources have increased to almost 20 million tons between 2012-2013, when compared to 11.8 million tons between 2004-2005.^13^ Processed food market in India is boosted mainly by wide variety of supply side factors such as water availability, low cost labor, agro climatic conditions favorable for wide variety of crops, product manufacturing enhancements, packaging technology enhancements and large live-stock base.^13^ Policy measures have significant facilitating effect which includes: 100% exclusion from taxation for period of five years, which is followed by 25% exemption from taxation for another 5 years for newer companies; 100% allowance of export oriented units for selling 50% of their own products in domestic market; waiving import duty on capital goods and raw materials for all the export oriented units; exemption of export goods from taxation and automatic route for 100% foreign direct investment.^14^ The above mentioned policy measures have helped mostly in the growth of sectors related to production of biscuits, chocolates, confectionary, savory snacks and ice creams. All these items are rich in fat, salt and sugars.

Departments under different ministries in India, either directly or indirectly linked to fat, salt and sugar markets, have stimulated a pro investment environment for these sectors without realizing the potential public health consequences.^13^ These policy initiatives further influence the behavior of people to consume more items rich in fat, salt and sugar.

## Public health effect of fat taxation

Increasing the taxes for unhealthy junk foods should encourage the people to take healthier food options like fruits and vegetables especially among younger adults.^15^ This has been proved bya systematic review conducted in 2013 by Powell et al who has reported that the 20% taxation on sugar sweetened drinks might reduce its consumption among younger adults by about 24%.^16^

Lancet Taskforce study, official partner of WHO Independent High level Commission on Noncommunicable Diseases, focused on several countries including India have reported that higher taxation on junk food not only helps higher income groups but also helps lower income groups to lead a healthier life, and stay safe from non-communicable diseases.^17^

Apart from Lancet study, several other studies around the world have proved positive impact of fat taxation. Cabrera Escobar et al conducted meta-analysis on the impact of raised prices of sugar sweetened beverages which showed a reduction in the prevalence of obesity and overweight.^18^ Jensen and Smed^19^ found that the fat consumption in Denmark dropped by about 10% following the taxation in 2011 while study conducted by Smed et al showed that consumption has reduced by 4%–5%.^11^ These studies provide sufficient evidence to suggest that taxes on food items can help in changing the consumption patterns and internalize the associated negative externalities.

Apart from health, another important effect is its contribution to country’s economy. Lancet Taskforce study showed that the price policies will affect large number of high income rather than the low income households, and the absolute increases in expenditure involved will be the largest for high income households. This is because the prevalence of consumption and the expenditure on alcohol, soft drinks, and snacks increases consistently with the household income.^17^

Another benefit associated with the taxation is the generation of revenue which can be used for various health initiatives and programmes to prevent obesity, support improvement of nutritional status and food quality and encourage the practice of physical activity.^16,20,21^

## International experience

### Denmark

Denmark was the first country in the world to introduce fat tax on October 2011 with an aim of reducing the burden of cardiovascular disease. As per the regulation, any food item which contains more than 2.3% of saturated fat will cost an extra 16 krone (US$3) per kilogram.^10^ This intervention was short-lived as the Danish government repealed the taxation within a year on November 2012. They have found that the Danish shoppers have found ways to circumvent this controversial taxation by purchasing the taxed items across the border in Sweden or Germany.^11^ This repeal has revealed the potential challenges with the fat taxation in country.

### Europe, US, Canada and Mexico

The imposition of value added tax (VAT) in Canada and Europe and sales taxes in the United States on food has shown certainty that even smaller taxation can help generate higher revenue for the country. Researchers have estimated that the national excise tax of 1 cent per 12 ounce for soft drinks could amount to the revenue generation of 1.5 billion US dollars per year.^22^ The Mexican government also imposed taxes on sugar containing sweetened beverages in September 2013 and also sales tax on various energy dense food items to reduce the burden of obesity and other nutrition related morbidities.^23^ Berkeley city in California, USA has also introduced taxation of sugar sweetened beverages.^24^

## Indian experience

### Kerala

In 2016, Kerala became the first state in India to impose a fat tax on fast food items sold in branded restaurants. The 14.5% fat taxation was introduced with an aim to curb the growing addiction to junk foods.^25^ Initially, fat tax was started with the unhealthy food items as it brings the least resistance from the people. There was further plan to slowly scale it up and include sugary beverages. Since this tax was targeted at big corporate food chain restaurants, the low-income people were not affected by it.

## Challenges in introduction of fat taxation throughout India

### Taxation debate

Major debate during the imposition of fat taxation were whether this taxation intended to reduce the burden of obesity across the state or country and change the consumption habits of the population or to generate additional revenue for the government through taxation or both. In competitive market conditions, incidence of taxation depends on relative price elasticity of the demand and supply of the products that are being taxed. If the demand curve becomes price inelastic, then the tax imposition will result in smaller reduction of quantity demanded compared to that of price elastic demand curve. In addition, if the demand curve is more elastic than supply curve, majority of the tax burden will eventually fall on the supplier’s side rather than the buyer’s and vice versa. Regarding the supply side, producers of the taxed fast food may not fully pass on the entire burden of taxation to the consumers. John Nye in his paper The Pigou Problem argues that any tax collection which is determined using the size of negative externality, but not considering all regulations and transfers affecting equilibrium, will not tell us what the optimal tax will be.^26^

### Ethical problems

A major ethical issue raised for the fat taxation is the autonomy of people to have daily access to food at a reasonable cost that is affordable for all as well as the decision to choose either healthy or favorable food items.^27^ Now, an ethical question may arise that if restricting particular food products from the consumers through taxation is ethically acceptable or not.

### Decision on setting the fat tax limit

One of the major concerns is how much fat tax should be set if planned for nationwide introduction. Answer for the above question can be derived by the evaluation of information about the current level of per capita fat consumption and the daily recommended level of fat consumption of the state/country in question as it can vary across different countries. Effects of input supplies, demand elasticity and substitution effects among different fat items should also be taken into consideration before setting the tax limit.^28^ However, the fat tax introduced in Kerala did not have sufficient work as mentioned above to justify the 14.5% taxation.

## Recommendations

### Subsidies on healthy food items

Fat taxation on their own will not have significant impact on the population’s consumption habits. Fiscal interventions are required to form an extensive strategy in public health nutrition which is more effective than the sole intervention. Hence, a fat tax should be implemented along with subsidy on unsaturated fatty acid items, fresh fruit and vegetable to promote healthy consumption habits.^29^

### Public health awareness

Creating awareness among the public regarding the consequences of consumption of unhealthy food items with excess saturated fats, transfats and sweeteners is important as it will help in justifying the fat taxation as well as causing further reduction in consumption of unhealthy food items. Studies have also shown that public health awareness activities and improving the nutrition related knowledge can significantly improve the consumption of healthy foods irrespective of an individual’s income level. Hence, successful public health campaign and programs should be conducted along with introduction of fat taxation throughout the country.

### Accessibility to healthy foods

Better accessibility to healthy foods may play a pivotal role in minimizing the habit of consuming unhealthy food items. Studies have also indicated that ease of access to healthy food items may shift the focus of individuals and change their dietary behaviors toward consumption of fresh vegetables and fruits.

### Health and nutrition promoting environment in school and workplaces

People spend most of their time in schools, colleges and workplaces and hence interventions should be targeted at such areas. Provision of nutrition education and availability of healthy snacks or food items in schools, childcare centers, colleges and other educational institutions, workplaces, hospitals can help in influencing healthy environment among children and adults.

## Conclusion

Nationwide implementation of fat tax might be beneficial in reduction of burden of obesity and also generate additional income for the country. However, fat tax alone cannot have significant impact on reducing non-communicable diseases. Hence, it should be coupled with effective public health awareness activities using the income generated through the taxation.

## Ethical approval

Not applicable.

## Competing interests

The authors declare that they have no competing interests.

## Funding

None.

## Authors’ contributions

All the authors were involved in designing the research, conducted research, extracted data and wrote the manuscript. All authors had primary responsibility for the final content of the manuscript and all authors read and approved the final manuscript.
